# Ill-Informed, Uplifted, and Unaware: A Psychiatrist Lives With a Condition Under His Purview

**DOI:** 10.7759/cureus.21007

**Published:** 2022-01-07

**Authors:** Andrés Martin

**Affiliations:** 1 Child Study Center, Yale School of Medicine, New Haven, USA

**Keywords:** psychiatry, autobiographical case report, mood disorders, lived experience, physician mental health

## Abstract

As part of a series of autobiographical case reports about physicians reporting on their own medical afflictions, a psychiatrist reflects on his lifelong experience with an illness under his discipline’s purview.

## Introduction

That I may rise, and stand, o'erthrow me, and bend your force, to break, blow, burn, and make me new. *― John Donne: Holy Sonnet XIV (1633)*

This many summers in a sea of glory

My career in medicine has been linear. My life in psychiatry seems preordained. My commitment to children and to words a natural return to the source.

Medicine was not the initial plan; philosophy was, whatever *that* was. As a child with severe myopia from a young age, I had two options: look at the written page or look inward. I became expert at both.

That inward gaze, combined with the place and time of my birth (Mexico City, 1966), made me fall in love with the written word. How could I have not, marinated as I was in regular installments of the Latin American Boom? The inchoate notion of philosophy proved too ethereal and abstract. But my backup plan was clear, tangible: I would write the next great (Latin) American novel. The problem was, much as I loved the idea, I hated writing. (Like Dorothy Parker, I have come to love "having written" [1].)

I was approaching high school graduation and needed a new plan, fast. With no college or liberal education opportunities back home, I had to put my chips down somewhere. I had to commit to a profession. Raised in a non-observant Jewish home, I nevertheless respected tradition: "Study Torah, but learn how to fix shoes." And so, philosophy begat literature, which begat medicine and psychiatry. Abstract Torah begat the practical and tangible shoemaking of medicine. My family sighed in relief and off I went.

I thrived in medical school. I enjoyed learning arcana I realized I'd never use. Among my muscular favorites: *origin, insertion, innervation, irrigation*. I knew before the first day of class that I would become a psychiatrist. My naïve notion was that the work would be akin to reading people’s stories. In the flesh, as it were. What was psychiatry if not applied humanities?

My choice of discipline was fated, obvious in retrospect. Determined by more than a love of personal stories and words, I had a stake in it. It was personal. Familial. My mother was ill for much of my life, not that we used the term or talked about it much. Under the cloak of her beauty and worldly, polyglot sophistication, she has whisked away to destinations unknown more times than I can recall. Even if unspoken, her depression became hard to miss. And unspoken *she* was. For over a decade, she lost her voice, or had a raspy residue of it. Surviving the Holocaust as a young woman in *Mitteleuropa* could not but leave emotional scars, whether obvious or silent.

It may have been less a desire to cure than to understand and decipher her, to listen to what she might say. But it was also out of the profound recognition of our likeness: it takes one to know one. My record-setting myopia was not the only gift I inherited from her. (I use the word "gift" deliberately and with no hint of sarcasm, something I will return to.)

I gravitated to patients with mood disorders, with whom I resonated. I became an expert of sorts, through books as much as personal experience. My first few episodes had gone unnoticed, unlabeled, unrecognized-untreated. By the time I knew better, I discovered that to my great fortune, medication worked. As did psychotherapy, those other strung-together words that proved a salve. Not only did I make my peace with depression: we formed a lifelong partnership. 

Was I depressed, or did I have depression? Was I mentally ill, or did I have a mental illness? Did "psychiatric illness" describe my predicament? Was depression my adjective or my noun? The syllable was difficult to separate from the sound. I may have questioned the labels or the identity but had developed a broad vocabulary to describe living the condition.

An acquaintance with depression at a young age and a love for words at an earlier one still: child psychiatry and intimacy with words returned me to my old haunts. I recognized fellowship in my young patients, rejoiced as a writer of modest contribution, a grateful reader, a meticulous wordsmith, and an editor immersed in words for a decade, fully engaged in a world of wordcraft [2].

I graduated from medical school at 24, was a certified child and adolescent psychiatrist by 29, a full professor at a world-famous institution by 41, and an endowed chair and editor-in-chief of my field’s flagship journal at 42 [3]. My enchanted career in medicine was as stepwise and linear as it gets. It was also free of sentinel events for those under my care or for me personally. My life in medicine was blissfully uncomplicated, predictable even. Until it was not.

## Case presentation

Far beyond my depth: my high-blown pride at length broke under me

*Frangere*, to break, is the Latin root for both fracture and fragility. Stress breaks us at fragile places―those we have come to know through aches, pains, or prior experience. The pandemic that started in late 2019 broke many. It broke *me*, though not immediately nor along recognizable fault lines. The pandemic broke so many of us in places unknown.

I have had a lifelong familiarity with depression. I have taught and written about mood disorders for most of my professional life [4-9]. I have shared my experiences in public in an effort to help others [10-15]. I literally wrote the book on it (edited it, at least) [16-18]. But for all my expertise, I did not see it coming. For that, I needed experiential learning, embodied acquaintance.

The lockdown and its interminable aftermath resulted in a blending of home and work lives, of weekdays and weekends, of sleep and wake cycles. With fewer colleagues working face-to-face at the inpatient unit that has been my primary professional home for the past two decades, a small cadre of us continued clinical work at a brisker pace. I cherished the opportunity to see patients and colleagues in person. It was as exhilarating as it was exhausting.

I was academically more productive than ever before. Videoconferencing opened entirely new venues for my research. The newfound efficiency of attending commute-free meetings freed up sorely needed time. Enforced confinement with no end in sight facilitated Dorothy Parker’s other pithy formula: “Writing is the art of applying the ass to the seat.” Paradoxically, a time of inconceivable loss and disruption turned out to yield a high watermark for original and innovative work.

It was in the same week that I received alerts from two entirely different sources, one at work, the other at home. My humor was too raw, too risqué. My jokes fell flat and were embarrassing. I was loud. I would not be quiet. I was restive. I became hard to follow. My non sequiturs were hard to keep up with. A colleague first noticed it at work, concerned that I could get into trouble, courtesy of my overly explicit and provocative language. What did she know, the alarmist? By the time a second sentinel, my dear wife, sought to prevent an impending collision, I had a flash of recognition: I was in the throes of my first hypomanic episode.

I ran to my psychiatrist. I knew what was at stake. In retrospect, the reduced need for sleep, racing thoughts, and distinct changes in mood were easy enough to recognize. I still had sufficient insight to know this did not bode well. Hypomania rings of smallness, a disease in a minor key. Bipolar II reeks of second place. I gladly accepted the runner-up status. I did not want to drop the hypo out of my mania: this initial acquaintance would more than suffice. With a revised diagnosis, treatment was straightforward, algorithm-driven: goodbye to a lifetime of antidepressants, hello to lamotrigine, my mood-stabilizing maiden voyage. My response was robust and prompt. I was all better. The episode lasted a few short weeks. I was good to go.

If only I had been.

Words reflect our experience as much as they give shape to it. But this time I had unusual difficulty finding words to express what I had lived through. Words for my internal experience were limited, opaque. I have developed by now a chromatic range to describe the subtle shadings of depression. This time around the terms were clumsy and dull. I was rendered mute. Hypomania was *wordless* under my insufferable verbosity, an irony that does not escape the son of his erstwhile *speechless* mother.

Irritability and raw emotion are convenient first approximations. On edge, high strung, fragile, exuding effect, conducting more voltage than designed to carry. I was getting to know the delicious and harrowing poison. My senses were oversharpened. Music was jarring, colors so vibrant I needed the dimmed indoors. Ansel Adams is credited with saying, "It is so beautiful it hurts." Which gives me the closest approximation I have yet found for the internal experience of hypomania: It is so beautiful it hurts―*too much*.

I continue struggling to find words to describe the experience, the emotional dimension of what ailed me. My use of the past tense is more hopeful than factual here. More than once I thought myself safely delivered from the straits, only to find myself overtaken by the elusive tidewater. The casualties left in its wake were easy to identify. The inward manifestations of the illness may be invisible; its outfacing repercussions are anything but.

## Discussion

Mercy of a rude stream

Depression has always been subtle in its advance. It insinuated itself slowly, imperceptibly. It was too late by the time it had slipped in and overtaken me. Paralysis and rumination heralded its arrival. Undetected, the indolent invasion took months; two full years during its last visitation.

Hypomania was different, my still inflated self-diagnostician opined. It had started and ended on distinct dates, discrete enough to pinpoint on a calendar. Two weeks, three at most, before the bucking bronco was lassoed back into submission [19].

My sense of timing was way off, perhaps another symptom of the hitherto unfamiliar ailment. Or perhaps simply a wish to encapsulate, if not to excise the lesion altogether. As it turns out, the time horizons were much broader on each end. On this proximal shore, I hesitate to consider myself on firm ground yet. Each time I believe the thing over and done with, I find its remnants in a supercharged and tearful, painfully raw self. Through this entire journey, I have not come across even a hint of depression, making the seascape all the more disorienting.

But it is on that other distant shore where I saw the real damage. A flitting and distractible mind that skipped more than one beat resulted in omissions with dire consequences, though none of them patient-related, as my clinical focus did not waver. A trail of social casualties stretched back many months before the purported start date. A bellicose cockiness that invited confrontation where there was none to have. An all-knowing arrogance alien to me.

It took the better part of the year following my diagnosis to mend what I had torn. I was well acquainted with guilt: the feeling comes naturally. But I was unfamiliar with shame. Hypomania schooled me quickly. The running list of those I confronted, wronged, hurt, or otherwise embarrassed grew to over a dozen. With my heart on my sleeve, I reached out to each one. I did not expect much. I had been awful, but what else is new in the battlefield of academia? Fessing up to a new diagnosis could be too facile a way to weasel myself out of responsibility for my actions: "It was not me; it was the unwelcome visitor." I did not want to excuse myself out of it. I was ready to face the music, to take my licks, to be stonewalled if I must.

I found varied responses, from shock and compassion to surprise at making so much over what some saw as so little. One of the colleagues I had let loose on considered my behavior nothing more than a standard academic skirmish: no expectation of an apology on his part. But I insisted. Even if he did not need to receive it, I could not exorcise my hurt and move forward without asking for his forgiveness. He recognized my plea for what it was and indulged me.

What I received was more than twelve indulgences. I was gifted with understanding and grace. I was forgiven, even before the requests left my lips. I found magnanimity and generosity of spirit I had not known before. I continue to be supported as I discover and organize the flotsam and jetsam lying in the wake of my whitewater river ride. It could have been so much worse. I did not spend or gamble my savings away. I did not embarrass myself in public nearly as much as I could have. I did not engage in sexual or romantic indiscretions that could have caused irreparable damage to the wife and four children I adore. I did not end up hospitalized. I did not graduate to first place, Número Uno bipolar disorder. Glory be.

I have ventured, Like little wanton boys that swim on bladders, This many summers in a sea of glory, But far beyond my depth: my high-blown pride At length broke under me and now has left me, Weary and old with service, to the mercy Of a rude stream...

The last line, from *Henry VIII* (III.2), first caught my eye as the title of a book I have come to love [20], the rueful recollection of an old literary lion making peace with ghosts past. I will take succor and guidance from wherever I can find them―lines from Shakespeare, an apt prescription, those standing steadfast by my side. I am profoundly grateful. I am at ease. I am at peace.

## Conclusions

I may well sense the tide when it next rises: I have gone from ill-informed to illness informed. I have revised my diagnosis but am not ill-formed. I was unaware but am not unwilling. I was uplifted but am not undone. I may have been bent but did not ultimately break. I would not give away two of the great gifts my mother gave me: I cannot conceive of myself without myopia or mental illness. I would not want to live without either one: it could be a good life no doubt, but it would not be mine.
